# Spatiotemporal hydro-chemical and isotopic dataset of the tropical Nyando river basin in Kenya

**DOI:** 10.1016/j.dib.2021.106787

**Published:** 2021-01-21

**Authors:** Benjamin Nyilitya, Stephen Mureithi, Pascal Boeckx

**Affiliations:** a Isotope Bioscience Laboratory - ISOFYS, Department of Green Chemistry and Technology, Faculty of Bioscience Engineering, Ghent University, Coupure Links 653, 9000 Gent, Belgium; b Department of Land Resource Management and Agricultural Technology, University of Nairobi, P. O. Box 29053-00625, Nairobi, Kenya; c National Water Resources Department, Ministry of Water & Sanitation and Irrigation, P. O. Box 49720-00100, Nairobi, Kenya

**Keywords:** Nitrate, δ^15^N‒NO_3_^−^, δ^18^O‒NO_3_^−^, Hydrochemistry, Source apportionment, Tropical basins

## Abstract

This article presents hydro-chemical and isotopic (δ^15^N-, δ^18^O‒NO_3_^−^, δ^11^B**)** data of water samples and potential nitrate sources from the Nyando river basin, a tributary of the Lake Victoria in Kenya. The data collection involved field sampling of water samples in 23 sampling stations spatially distributed in the basin during nine seasons from July/2016 to May/2018. The hydro-chemical data was generated from the Laboratory analysis of the water samples using the ion chromatogram. Samples for nitrate isotope (δ^15^N-, δ^18^O‒NO_3_^−^) analysis were prepared via the bacterial denitrification method and analysed using Isotope Ratio Mass Spectrometer. The data, which is categorised in different land use zones and seasons, is important for understanding the spatiotemporal variation in nitrate and solute concentrations and the role of land use on the river water quality. In addition, the δ^15^N-, δ^18^O‒NO_3_^−^ and δ^11^B values are key for elucidating nitrate pollution sources and potential biogeochemical processes for the management and control of nutrient pollution and eutrophication of the Lake Victoria. Furthermore, the dataset can be of great use in water quality models for understanding non-point pollution dynamics in tropical basins. This article is related to [1].

**Specifications Table**

**Table:** 

Subject	Environmental science
Specific subject area	Hydrology and Water Quality
Type of data	Table
How data were acquired	Laboratory determination of hydro-chemical parameters (Na^+^, K^+^, Ca^2+^, Mg^2+^, NO_3_^−^, NO_2_^−^, Cl^−^, and SO_4_^2−^) concentrations was carried out using an ion chromatogram (930 Compact IC Flex, Metrohm, Switzerland). Determination of water temperature (T), electrical conductivity (EC), pH, and dissolved oxygen (DO) was done during field sampling using a multi-parameter sensor (2FD47F-Multi3430, WTW, Germany). On the other hand, sample preparation for δ^15^N‒ and δ^18^O‒NO_3_^−^ analysis was performed via the “Bacterial denitrification method” [2], [3], [4], and the δ^15^N and δ^18^O analysis carried out using a trace gas preparation unit (ANCA TGII, SerCon, UK), coupled to an isotope ratio mass spectrometer (IRMS) (20-20, SerCon, UK). The subsequent stable isotope data were expressed as delta (δ) units in per mil (‰) notation relative to the respective international standards: $\delta_{sample}\left(\%\right)=\left[\frac{R_{sample}}{R_{standard}}-1\right]\times 1000$ (1) Where $R_{sample}\;$ and $R_{standard}$ are the ^15^N/^14^N or ^18^O/^16^O ratio of the sample and the standard for δ^15^N and δ^18^O, respectively. δ^15^N values are reported relative to N_2_ in atmospheric air (AIR) and δ^18^O are reported relative to Vienna Standard Mean Ocean Water (VSMOW). The water samples and potential source (end member) analysis technique for B concentrations and δ^11^B values is well covered by [5]. Similar to δ^15^N-, δ^18^O‒NO_3_^−^, B isotope ratios were expressed in delta (δ) units and a per mil (‰) notation relative to an international standard, NBS951. The data is presented in tables generated using Microsoft Excel 2013 software.
Data format	Raw Analysed
Parameters for data collection	During field sampling, river water samples were pre-filtered onsite using 11 µm filters (Whatmann, GE Healthcare Life Sciences, Chicago, IL, USA) and stored in an insulated cooler box containing ice cubes so as to keep a constant temperature of around 4 °C during transportation to the laboratory. Samples for cation analysis were acidified (after pre-filtration) to pH 2 using diluted hydrochloric acid. In the laboratory, all samples for δ^15^N‒ and δ^18^O‒NO_3_^−^ analysis were filtered again through 0.45 µm membrane filters and stored frozen (-17°C) awaiting analysis. Isotope results were only accepted if measured δ^15^N and δ^18^O values of the laboratory standard were within 0.4 and 0.5 ‰ of our accepted values, respectively. If standard deviation on replicate samples was higher than 0.3 and 0.4 for δ^15^N and δ^18^O, respectively, the sample was reanalyzed.
Description of data collection	The data presented is for 23 spatially distributed stations in the Nyando river, in addition to data for the potential sources of nitrate pollution in the basin. Sampling campaigns were contacted during four seasons within an hydrological year: (1) the transition period between dry and wet season in March, marked as ‘start wet’ season (SW); (2) the agriculturally productive wet period between May – July, marked as ‘peak wet’ (PW); (3) during the ‘end of the wet season’ (EW) in September; and (4) in the dry season (D) in December as described in [6]. This was contacted for nine seasons (i.e. 2 SW, 3 PW, 2 EW, and 2 D) from July 2016 to May 2018.
Data source location	The data is from the Nyando river, which drains into the Lake Victoria, Kenya. See the GPS points in Fig. 1 and Table 1. Isotopic variables were analysed at the Isotope Bioscience Laboratory (ISOFYS), Department of Green Chemistry and Technology, Faculty of Bioscience Engineering, Ghent University, Gent-Belgium Hydro chemical variables were analysed at the Department of Land Resource Management and Agricultural Technology, University of Nairobi, Nairobi-Kenya
Data accessibility	With the article
Related research article	B. Nyilitya, S. Mureithi, M. Bauters, P. Boeckx, Nitrate source apportionment in the complex Nyando tropical river basin in Kenya, J. Hydrol. 594 (2021) 125926. https://doi.org/10.1016/j.jhydrol.2020.125926

## Value of the Data

• The data is important for revealing nitrate pollution sources and potential biogeochemical processes for the management and control of nutrient pollution sources in tropical sub-Saharan African (SSA) river basins
• The physicochemical dataset is quite useful in understanding water types, origin and processes governing dissolution of solutes in the basin.
• This data is key to researchers, managers and policy makers involved in water and environmental resources management especially in the tropics.
• Similar isotope data (δ^15^N-, δ^18^O‒NO_3_^−^ & δ^11^B) is rare in tropical SSA river basins. Therefore, further analysis of this pioneer dataset can give new ideas for future research in the region.
• The geo-referenced dataset containing hydro-chemical and isotopic variables is quite applicable in water quality modelling for deciphering non-point pollution dynamics in tropical basins.

## 1 Data Description

**Fig. 1:**
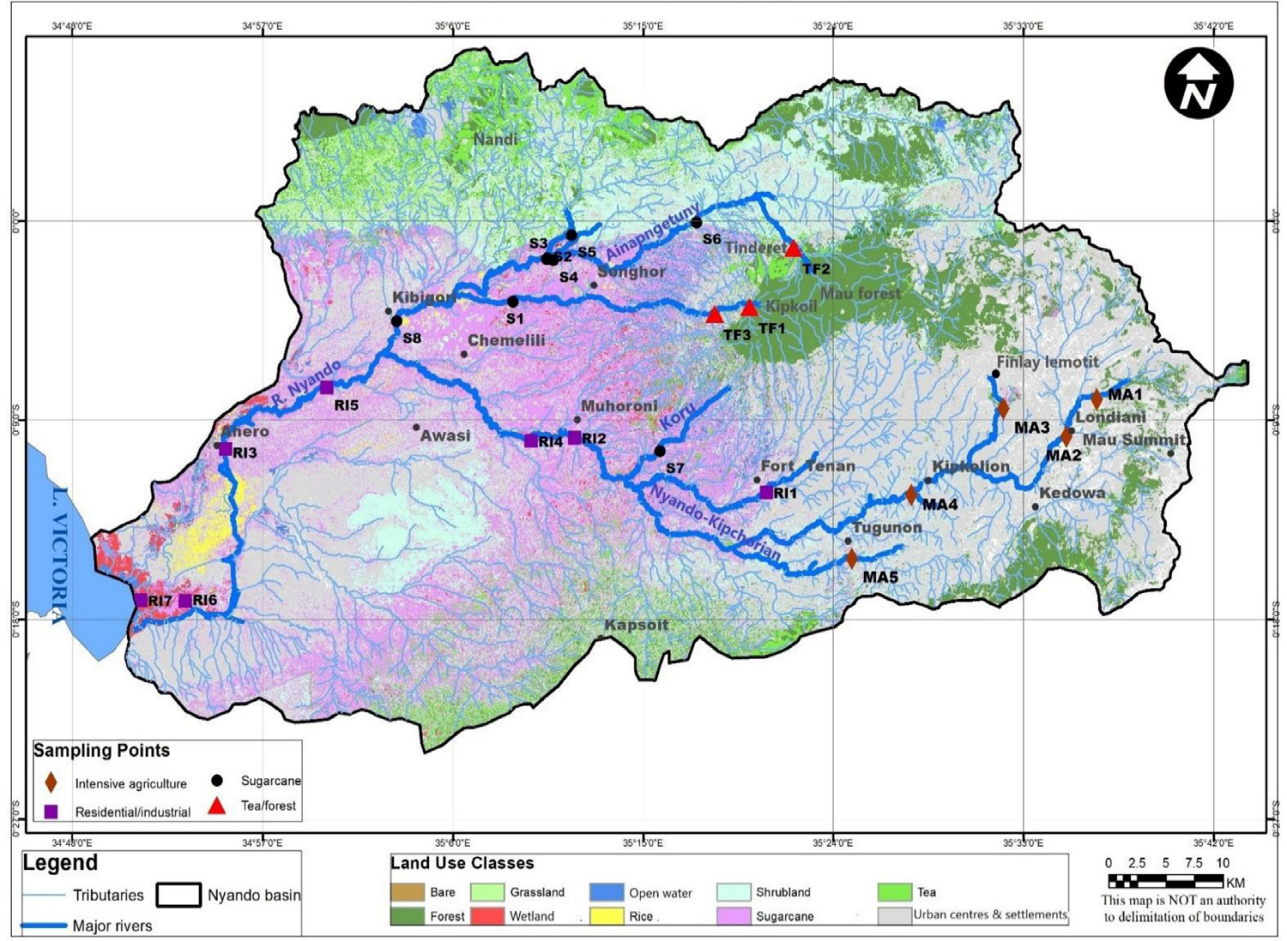
Map of the Nyando river basin. Spatial sampling stations are labelled using bullets which represents the dominant land use characteristics of the basin, MA1 – MA5 (diamonds): mixed agriculture, RI1 – RI7 (squares): residential & industrial, S1 – S8 (circles): Sugarcane, TF1 – TF3 (triangles): tea & forests. Source: [1].

The data presented consists of hydro-chemical (Na^+^, K^+^, Ca^2+^, Mg^2+^, NO_3_^−^, NO_2_^−^, Cl^−^, SO_4_^2−^, T, EC, pH, DO) and isotopic (δ^15^N‒, δ^18^O‒NO_3_^−^ and δ^11^B‒B) parameters of water samples from the Nyando river basin in Kenya obtained during nine seasons of field monitoring. The spatial distribution of the sampling stations in the basin is presented in Fig. 1. Through the analysis of the hydro-chemical parameters via Hierarchical Cluster Analysis (HCA), the spatial stations grouped into distinct clusters which match the four main land use characteristics of the river basin. These are: Mixed Agriculture (MA), Residential & Industrial (RI), Sugarcane (S), and Tea & Forest (TF). The labelling of sampling stations in Fig. 1 is based on these land use clusters. For purposes of investigating nitrate pollution sources, nitrate and boron isotope data of the potential nitrate sources in the basin are presented in Table 4.

**Table 1:** The spatial distribution of NO_3_^−^ concentration (mgL^−1^) in R. Nyando during the nine season monitoring period (2016 – 2018). Values < 0.04 indicate attributes below detection limit; “‒” represents samples not analyzed.

Land use	Station ID	Lat.(S)	Long.(E)	Peak wet/2016	End wet/2016	Dry/2016	Start wet/2017	Peak wet/2017	End wet/2017	Dry/2017	Start wet/2018	Peak wet/2018
Mixed Agriculture	MA1	0.135	35.608	7.6	10.4	8.1	6.1	5.8	7.3	9.9	6.9	4.1
	MA2	0.163	35.584	6.4	5.6	7.0	4.4	4.3	5.4	7.1	4.9	4.5
	MA3	0.142	35.534	5.6	11.6	23.8	22.9	23.7	26.9	44.2	56.3	4.1
	MA4	0.207	35.462	4.0	3.1	2.5	0.6	7.5	6.2	4.5	5.7	7.1
	MA5	0.255	35.415	2.3	1.0	1.0	8.5	5.8	10.5	4.4	5.0	9.1
Residential & Industrial	RI1	0.204	35.348	1.7	3.8	1.0	1.1	3.5	5.9	4.1	5.8	6.7
	RI2	0.163	35.196	3.3	3.4	0.4	1.0	5.7	7.5	1.0	7.3	6.7
	RI3	0.172	34.921	1.4	1.8	0.2	3.2	4.9	5.5	3.2	5.1	5.2
	RI4	0.166	35.162	3.6	2.6	0.1	<0.04	5.2	7.2	2.1	6.7	7.3
	RI5	0.126	35.001	1.5	3.4	0.5	0.9	4.3	5.2	2.4	6.4	4.2
	RI6	0.286	34.889	2.8	1.1	0.3	2.8	2.9	5.6	1.1	5.1	3.8
	RI7	0.286	34.854	1.9	2.0	0.2	2.3	2.6	5.5	3.3	4.2	2.2
Sugarcane	S1	0.099	34.751	2.4	0.3	0.1	<0.04	2.5	4.4	2.0	3.2	2.9
	S2	0.028	35.175	3.4	1.3	1.5	0.4	4.0	5.6	4.1	4.1	7.4
	S3	0.029	35.174	3.1	2.7	1.4	0.6	3.4	4.5	3.3	3.5	3.4
	S4	0.030	35.179	2.6	2.4	1.4	1.0	3.0	4.1	3.0	3.3	5.3
	S5	0.011	35.194	1.6	2.3	1.2	0.9	3.0	4.7	3.8	4.1	6.7
	S6	0.001	35.292	3.5	2.7	1.9	1.8	3.3	4.0	3.5	2.7	5.4
	S7	0.173	35.263	2.5	1.2	1.4	0.4	2.7	4.8	2.3	6.2	6.3
	S8	0.076	35.056	‒	‒	2.1	0.8	3.1	4.6	1.5	3.5	4.7
Tea & Forest	TF1	0.065	35.334	3.3	1.9	1.9	2.9	3.6	4.0	4.5	2.3	4.1
	TF2	0.021	35.369	2.5	2.9	1.6	2.1	2.0	4.4	2.8	1.6	4.2
	TF3	0.070	35.307	3.8	2.5	1.6	2.5	4.3	<0.04	4.2	2.6	5.2

**Table 2:** The spatial δ^15^N‒NO_3_^−^ values (‰) in R. Nyando during the nine season monitoring period (2016 – 2018). “‒” represents samples not analyzed.

Land use	Station ID	Peak wet/2016	End wet/2016	Dry/2016	Start wet/2017	Peak wet/2017	End wet/2017	Dry/2017	Start wet/2018	Peak wet/2018
Mixed Agriculture	MA1	9.6	8.1	8.6	8.9	8.8	8.3	11.2	10.2	10.8
	MA2	9.5	9.8	8.8	8.9	10.0	7.8	10.7	11.6	10.4
	MA3	14.0	9.0	9.5	13.9	13.7	10.1	11.8	10.7	12.9
	MA4	8.8	6.8	12.4	9.4	9.7	7.2	10.3	11.1	11.4
	MA5	11.8	7.3	4.8	5.3	12.1	7.0	‒	13.8	13.6
Residential & Industrial	RI1	10.3	8.4	10.9	11.8	10.5	7.4	10.1	11.2	11.3
	RI2	8.9	9.4	12.1	8.2	10.0	6.9	‒	11.4	11.4
	RI3	6.3	7.5	8.3	8.2	10.0	5.1	‒	11.3	11.6
	RI4	9.3	8.6	8.2	‒	9.9	8.2	8.9	9.8	11.9
	RI5	8.0	9.0	15.1	7.8	7.4	6.5	13.1	9.1	11.2
	RI6	9.2	7.1	11.1	9.2	7.5	14.4	‒	10.4	11.2
	RI7	8.6	8.8	8.6	9.3	7.2	8.5	‒	11.3	11.9
Sugarcane	S1	7.4	7.9	9.8	‒	6.9	7.5	‒	8.6	8.8
	S2	10.0	8.6	8.6	9.0	6.6	6.8	‒	9.5	9.7
	S3	8.8	6.1	10.4	8.8	7.0	6.7	‒	10.0	9.4
	S4	9.1	8.1	8.5	9.4	6.5	7.1	‒	9.8	8.9
	S5	10.0	7.6	9.4	7.3	7.3	6.7	‒	10.0	9.3
	S6	9.2	9.1	8.6	9.6	8.5	6.4	9.9	11.3	9.2
	S7	10.2	10.5	11.8	9.6	8.5	6.8	8.8	9.2	9.8
	S8	‒	‒	7.8	8.6	7.7	11.9	‒	9.5	10.6
Tea & Forest	TF1	7.4	5.4	6.6	8.8	6.7	5.5	9.1	9.0	7.4
	TF2	5.1	3.8	7.2	6.4	5.6	‒	6.3	7.2	6.4
	TF3	6.4	7.0	8.9	9.0	7.9	‒	‒	9.8	7.8

**Table 3:** The spatial δ^18^O‒NO_3_^−^ values (‰) in R. Nyando during the nine season monitoring period (2016 – 2018). “‒” represents samples not analyzed.

Land use	Station ID	Peak wet/2016	End wet/2016	Dry/2016	Start wet/2017	Peak wet/2017	End wet/2017	Dry/2017	Start wet/2018	Peak wet/2018
Mixed Agriculture	MA1	11.5	5.1	11.0	3.4	5.5	8.5	6.3	6.8	4.0
	MA2	9.4	11.8	10.2	2.3	4.0	11.1	6.2	8.2	7.0
	MA3	19.6	11.1	16.6	17.0	17.0	17.3	17.8	16.3	13.4
	MA4	10.3	2.4	11.7	1.0	7.2	8.8	6.5	7.1	8.2
	MA5	9.1	-1.1	-4.5	9.1	8.6	6.2	‒	6.9	12.8
Residential & Industrial	RI1	10.7	6.9	9.3	6.0	6.0	6.4	5.4	5.7	10.0
	RI2	9.1	8.8	8.9	3.4	7.5	9.0	‒	6.4	7.7
	RI3	12.0	5.8	1.6	8.6	6.3	7.3	‒	6.0	9.9
	RI4	10.5	8.1	12.5	‒	7.6	7.2	3.6	4.1	10.5
	RI5	8.4	3.1	7.2	0.2	10.0	14.9	14.9	5.1	7.5
	RI6	11.4	4.5	4.8	4.4	10.3	12.9	‒	4.4	10.9
	RI7	10.6	6.3	1.4	4.4	11.4	11.0	‒	6.9	11.6
Sugarcane	S1	7.9	12.7	3.8	‒	6.6	5.1	‒	5.3	7.0
	S2	8.4	9.6	9.7	9.1	7.3	6.7	‒	7.0	5.1
	S3	6.8	9.0	10.2	5.5	5.4	11.4	‒	4.8	5.6
	S4	6.2	8.7	7.6	5.9	7.5	5.8	‒	6.8	7.6
	S5	9.0	6.2	2.8	7.1	6.0	6.0	‒	5.3	6.4
	S6	8.8	8.5	9.7	6.2	8.1	7.2	10.1	4.9	5.4
	S7	12.5	8.0	15.6	5.5	6.7	7.2	4.7	6.5	8.0
	S8	‒	‒	7.0	5.2	6.5	15.2	‒	5.7	10.7
Tea & Forest	TF1	8.6	4.1	5.7	4.8	7.3	7.1	-0.3	6.0	4.0
	TF2	5.0	3.4	10.6	4.7	4.4	‒	0.6	5.6	6.0
	TF3	6.1	6.6	12.0	5.0	5.3	‒	‒	5.2	5.0

**Table 4:** Boron concentration and isotopic (δ^11^B, δ^15^N-NO_3_^−^, δ^18^O-NO_3_^−^) values of potential nitrate sources of the Nyando basin in Kenya. “‒” represents samples not analyzed.

Sources	Sample ID	B	δ^11^B	δ^15^N-NO_3_^−^	δ^18^O-NO_3_^−^
Manure	CM	127	37.0	9.5±4.9	6.5±1.0
	FM	181	36		
	LCM	438	11		
	BCM	148	37		
	GSM	581	31		
Urban Sewage (liquid)	Kisat	46.3	16.2	14.9±6.1	16.8±4.9
	Auji	33.4	31.8		
	Kisat raw	26.6	18.9		
	Musco	25.0	22.0		
NO_3_^−^ Fertilizer	NPK	736	-4.3	2.5±0.2	24.0±2.0
	CAN	14.9	-1.5		
NH_4_^+^ Fertilizer	Urea	3.9	17.3		
	DAP	2500	7.8	(-)1.8±0.9	6.5±1.0
	DAP(b)	1050	52		
Soil N (10 samples)		-	-	1.7±0.2	6.5±1.0
Rainfall (4 samples)		-	-	2.1±6.3	41.8±20.6

**Table 5:** Spatial Nyando river hydro-chemical dataset for the nine season sampling campaigns: peak wet/2016, end wet/2016, dry/2016, start wet/2017, peak wet/2017, end wet/2017, dry/2017, start wet/2018, peak wet/2018. Values < 0.01 or < 0.04 indicate attributes below detection limit; “‒” represents samples not analyzed.

Season	ID	Na^+^	K^+^	Ca^2+^	Mg^2+^	Cl^−^	SO_4_^2−^	NO_2_^−^	pH (°C)	Cond	Temp	DO
Peak wet/2016	MA1	17.3	5.4	7.5	<0.04	5.4	2.4	0.02	7.3	147	15.2	7.3
	MA2	16.9	5.9	6.3	<0.04	9.3	3.9	0.04	7.2	150	15.8	7.0
	MA3	18.9	5.5	6.8	2.9	4.6	1.3	0.03	7.4	168	19.4	6.7
	MA4	19.5	6.8	8.6	1.8	8.2	4.4	0.06	7.8	180	18.8	7.4
	MA5	11.9	6.6	9.0	<0.04	5.9	2.9	0.04	7.8	153	16.5	7.6
	RI1	25.5	5.9	11.3	4.7	4.5	1.2	0.04	8.2	271	20.3	7.5
	RI2	21.7	5.9	10.8	3.4	6.2	2.3	0.03	8.2	216	22.5	7.3
	RI3	14.3	4.8	10.8	2.9	4.1	2.0	0.07	7.6	162	21.9	6.8
	RI4	21.5	8.5	11.7	4.0	7.0	3.1	<0.01	7.9	241	22.7	6.7
	RI5	16.2	6.6	13.5	4.1	5.0	2.0	0.04	7.8	215	20.3	7.9
	RI6	16.3	6.1	11.0	3.8	4.4	2.2	0.01	8.0	213	22.6	7.0
	RI7	17.0	6.1	11.8	3.8	5.0	2.0	0.01	7.9	216	23.0	6.9
	S1	8.4	3.1	11.8	6.7	1.7	0.9	<0.01	7.2	190	22.0	7.2
	S2	15.6	3.5	7.6	6.4	2.9	0.8	0.01	8.1	272	20.9	7.7
	S3	16.0	4.6	8.6	7.4	3.3	1.3	0.01	8.3	280	21.4	7.5
	S4	16.1	4.7	7.6	7.5	2.6	1.1	0.01	8.4	280	21.8	7.5
	S5	17.6	3.8	7.1	6.9	2.4	0.4	0.01	8.1	299	21.7	7.4
	S6	14.8	4.4	11.0	5.7	3.9	1.9	0.02	8.3	236	18.9	7.8
	S7	11.2	4.5	13.6	9.7	2.7	1.5	0.01	8.4	323	21.9	7.2
	TF1	7.3	1.5	10.2	5.4	3.2	1.5	0.06	7.8	147	16.1	7.7
	TF2	6.5	2.4	9.5	5.1	1.7	0.6	0.02	7.8	137	16.6	7.7
	TF3	5.9	2.3	9.0	5.6	2.7	1.6	0.01	7.8	148	19.6	7.2
End wet/2016	MA1	16.5	11.3	5.9	1.4	6.9	4.1	0.02	7.5	143	15.3	7.0
	MA2	17.2	11.0	8.3	1.9	8.9	4.6	0.06	7.5	167	14.6	6.8
	MA3	15.9	6.5	12.2	4.9	4.3	1.7	0.01	7.5	196	16.7	7.0
	MA4	25.3	8.6	9.6	2.5	5.3	3.0	0.02	8.3	225	18.2	7.4
	MA5	14.5	7.2	9.7	2.4	2.6	0.7	0.04	-	-	-	-
	RI1	20.2	7.0	12.4	4.3	5.1	2.6	0.02	8.3	232	19.6	7.5
	RI2	19.0	6.6	15.5	4.2	5.8	3.1	0.04	8.4	218	22.4	7.3
	RI3	17.3	10.7	12.7	5.3	5.1	2.7	0.13	7.8	247	22.5	6.7
	RI4	21.3	17.5	18.1	5.5	6.2	2.9	0.05	8.0	287	23.4	4.1
	RI5	-	-	-	-	5.1	2.5	0.03	7.9	259	21.4	7.4
	RI6	18.9	9.1	17.2	5.1	2.9	1.6	0.06	8.0	249	23.0	6.6
	RI7	17.8	9.8	13.8	5.4	3.8	1.5	0.06	8.0	258	23.6	6.6
	S1	5.6	2.6	12.4	6.0	1.4	0.9	<0.01	8.4	154	21.2	7.3
	S2	14.2	3.7	10.6	5.9	1.6	0.3	0.01	8.2	253	20.5	7.9
	S3	14.6	4.7	12.4	6.2	4.1	1.7	0.03	8.4	243	20.5	7.8
	S4	14.2	4.6	12.4	6.1	3.6	1.5	0.02	8.2	242	20.7	7.8
	S5	16.6	4.4	15.0	7.3	3.7	1.0	0.02	8.2	301	20.3	8.2
	S6	13.5	4.5	13.6	4.8	4.0	1.7	0.02	8.4	205	18.6	7.8
	S7	10.2	5.4	21.9	9.6	2.4	1.4	0.03	8.3	304	22.5	7.1
	TF1	5.6	2.4	11.6	5.6	1.4	1.0	0.03	7.9	137	16.2	7.7
	TF2	6.0	3.0	10.8	4.9	2.2	1.2	0.01	7.6	135	15.8	7.8
	TF3	5.8	2.5	13.3	5.9	2.2	1.9	0.02	8.1	153	18.2	7.5
Dry/2016	MA1	15.8	6.3	5.9	1.4	7.1	4.0	0.04	7.5	141	15.4	7.1
	MA2	16.5	7.0	6.6	1.6	7.3	3.3	0.11	7.7	152	15.5	7.2
	MA3	20.9	5.9	11.1	5.7	4.6	4.7	0.05	7.1	235	18.1	5.1
	MA4	33.6	10.7	8.1	2.6	9.3	5.1	0.04	8.6	267	22.1	7.0
	MA5	23.1	11.3	14.3	4.0	5.1	2.3	0.03	8.0	299	20.7	7.1
	RI1	35.7	11.6	16.4	9.4	6.0	1.7	0.01	8.4	424	21.5	6.7
	RI2	32.6	9.5	13.6	8.4	5.3	1.9	0.08	8.6	377	25.4	7.6
	RI3	26.1	19.8	19.7	10.1	5.8	2.0	0.16	8.3	401	22.6	7.7
	RI4	35.2	69.4	26.2	12.8	12.3	2.7	0.01	8.1	621	27.0	0.1
	RI5	24.7	20.4	18.5	9.8	5.6	1.4	0.26	8.4	389	23.3	6.8
	RI6	26.6	20.2	18.0	9.2	5.9	2.3	0.18	8.3	399	26.0	5.6
	RI7	26.8	20.2	18.6	9.6	6.4	2.3	0.09	7.9	412	25.7	3.5
	S1	9.5	3.4	23.4	10.3	1.8	0.7	<0.01	-	-	-	-
	S2	15.5	4.2	13.7	7.1	2.4	0.4	0.04	8.3	275	20.7	8.0
	S3	16.6	4.9	16.4	10.4	3.3	0.8	0.08	8.7	323	21.3	8.1
	S4	17.0	5.2	11.2	11.0	3.5	0.9	0.03	8.5	333	23.0	7.4
	S5	16.8	5.2	17.6	8.1	3.1	0.4	<0.01	8.4	330	22.9	7.1
	S6	15.7	4.8	11.4	8.4	3.9	1.1	0.04	8.5	285	19.0	7.7
	S7	15.3	7.2	18.5	14.0	2.5	1.0	<0.01	8.5	424	23.4	7.4
	S8	17.6	4.1	10.9	9.1	3.4	0.9	0.02	8.2	296	21.6	7.9
	TF1	6.9	2.5	9.4	7.6	1.5	1.5	0.11	8.1	193	15.3	7.7
	TF2	6.8	2.8	9.2	7.7	1.7	1.2	0.08	8.1	187	15.6	7.8
	TF3	6.6	2.6	10.3	7.5	1.5	1.3	0.1	7.9	186	17.9	7.3
Start wet/2017	MA1	14.9	6.4	6.3	1.5	6.0	3.0	0.05	5.1	114	18.4	6.0
	MA2	16.1	7.0	5.8	1.6	6.6	3.1	0.03	6.0	124	18.7	5.6
	MA3	21.3	7.2	9.2	6.4	3.1	1.6	0.5	5.8	221	18.9	6.0
	MA4	36.6	10.5	7.3	2.5	4.2	1.8	<0.01	6.1	254	24.0	6.2
	MA5	15.9	11.4	9.0	1.9	4.4	2.7	0.44	7.5	168	22.9	5.3
	RI1	47.2	9.9	8.5	9.7	4.3	1.1	<0.01	8.4	434	25.4	5.7
	RI2	30.8	8.4	9.6	7.3	3.7	1.2	<0.01	8.4	321	27.6	4.8
	RI3	15.7	12.9	12.7	5.3	3.5	3.2	0.21	7.8	240	25.3	4.4
	RI4	28.3	19.8	14.8	7.7	3.9	1.4	<0.01	7.8	476	27.9	5.3
	RI5	30.3	13.4	11.2	10.3	4.3	1.2	0.03	7.8	356	26.7	4.9
	RI6	21.2	16.6	12.5	6.4	4.7	2.7	0.04	8.0	299	27.3	5.7
	RI7	20.4	16.0	13.0	6.3	3.9	2.0	0.04	7.7	292	28.8	4.8
	S1	7.7	3.7	15.5	7.1	2.1	1.5	0.11	8.1	182	21.6	7.5
	S2	17.2	5.3	12.3	8.0	2.8	0.4	<0.01	8.2	280	22.4	5.4
	S3	16.0	5.5	9.8	11.9	1.4	0.3	<0.01	8.4	320	22.7	6.2
	S4	15.4	5.4	8.3	12.7	1.8	0.4	<0.01	8.5	328	23.0	5.8
	S5	19.2	9.0	13.4	8.0	2.9	0.4	<0.01	8.0	334	20.8	5.7
	S6	13.5	4.8	6.8	10.1	3.1	0.7	<0.01	8.5	288	20.5	3.2
	S7	17.0	8.0	13.1	14.4	1.4	0.5	<0.01	8.4	403	26.5	6.1
	S8	20.3	5.0	12.1	11.0	-	-	<0.01	8.2	307	26.0	5.5
	TF1	7.6	3.6	9.6	8.2	2.0	1.3	<0.01	8.2	194	16.6	4.5
	TF2	7.6	4.3	10.7	9.1	2.0	0.6	<0.01	8.1	206	18.0	5.0
	TF3	7.3	4.0	10.6	7.9	2.2	1.1	<0.01	7.8	188	20.8	4.3
Peak wet/2017	MA1	15.2	6.1	5.5	<0.04	7.1	3.6	0.06	7.4	141	17.2	6.6
	MA2	17.7	8.3	7.4	2.1	10.1	3.4	0.06	7.3	186	18.4	6.4
	MA3	13.8	3.7	5.6	3.6	1.9	1.9	<0.01	7.1	179	19.7	6.3
	MA4	22.6	9.1	8.5	2.2	8.0	6.1	0.06	7.4	217	22.7	6.8
	MA5	12.4	6.3	8.4	1.7	5.0	3.1	0.04	7.4	147	17.0	7.5
	RI1	28.7	8.4	13.4	7.2	5.8	3.3	0.03	7.5	339	21.4	7.3
	RI2	15.7	6.2	8.7	3.2	4.5	2.7	0.03	7.6	216	21.7	7.4
	RI3	14.2	5.7	8.5	3.4	3.5	2.5	<0.01	7.6	228	27.7	6.6
	RI4	17.1	7.1	10.1	4.8	4.2	2.6	0.04	7.4	224	22.2	7.0
	RI5	15.7	6.3	8.4	4.3	-	-	<0.01	7.4	231	25.1	6.9
	RI6	13.4	4.4	8.3	1.6	2.5	2.5	0.05	7.4	157	23.3	5.8
	RI7	22.5	3.5	7.8	1.4	2.0	1.8	0.05	7.5	172	23.5	5.7
	S1	8.4	3.1	11.8	6.7	1.7	0.9	<0.01	7.2	190	22.0	7.2
	S2	9.8	5.1	9.7	3.8	2.7	1.4	0.05	7.3	169	21.3	7.3
	S3	14.0	5.3	10.9	7.9	3.1	1.3	<0.01	7.6	275	22.0	7.3
	S4	14.5	5.1	12.3	8.3	3.0	1.1	<0.01	7.4	290	22.5	7.2
	S5	13.6	5.8	11.1	6.1	2.9	1.1	<0.01	7.3	266	22.0	7.1
	S6	14.8	4.8	14.4	8.8	3.8	1.5	<0.01	7.5	295	19.3	7.6
	S7	8.6	4.3	15.2	8.5	2.1	1.7	<0.01	7.3	284	20.7	7.4
	S8	14.1	4.1	10.6	6.6	2.8	1.6	<0.01	7.4	237	26.2	6.7
	TF1	4.6	2.2	9.1	5.1	1.3	1.6	<0.01	7.6	160	16.6	7.5
	TF2	6.6	3.1	6.2	7.6	0.9	0.4	<0.01	7.3	213	17.0	7.6
	TF3	4.6	2.3	10.6	5.6	1.4	1.4	<0.01	7.5	146	20.0	7.2
End wet/2017	MA1	16.2	6.9	7.5	1.4	9.6	6.7	0.05	7.8	144	15.0	7.0
	MA2	11.7	6.2	6.6	1.2	2.3	2.7	0.05	7.6	126	16.0	6.6
	MA3	14.6	3.9	12.5	4.8	-	-	<0.01	7.7	193	18.0	6.7
	MA4	15.1	6.8	8.3	1.6	5.7	5.5	0.05	8.1	145	18.7	7.4
	MA5	9.3	3.9	11.4	1.7	3.2	1.8	0.05	8.1	127	19.6	7.1
	RI1	14.2	5.2	12.8	3.4	3.4	3.0	0.04	8.6	190	19.0	7.6
	RI2	13.2	6.1	10.1	2.5	4.1	3.5	0.05	8.4	155	21.0	7.5
	RI3	13.1	5.5	11.2	2.5	3.6	3.2	0.08	8.1	150	20.1	7.3
	RI4	13.0	6.5	11.3	2.7	4.1	3.5	0.06	8.3	161	21.1	7.3
	RI5	11.2	5.5	9.5	2.4	3.3	2.9	0.06	8.1	142	20.1	7.7
	RI6	13.6	6.1	11.8	2.9	3.5	2.9	0.04	8.1	173	21.6	6.5
	RI7	13.5	6.2	12.0	3.1	3.5	2.9	0.06	8.0	174	22.2	6.0
	S1	5.6	2.6	12.4	6.0	1.4	0.9	<0.01	8.4	154	21.2	7.3
	S2	10.6	4.4	16.3	4.6	2.8	1.6	0.05	8.3	198	19.5	7.6
	S3	11.7	4.9	13.0	4.7	2.9	1.9	0.04	8.3	204	20.0	7.6
	S4	11.9	5.0	14.7	4.7	3.1	2.2	0.04	8.3	207	19.3	7.5
	S5	11.1	4.4	15.0	5.2	2.7	1.4	0.03	8.3	225	19.7	7.5
	S6	10.5	4.3	11.4	3.6	3.3	2.4	<0.01	8.4	196	17.5	8.0
	S7	7.7	3.9	19.9	7.9	1.7	1.7	0.04	8.5	248	21.8	7.2
	S8	10.4	4.2	12.1	4.1	2.7	2.2	0.05	8.3	171	20.3	7.4
	TF1	3.9	2.0	9.3	4.1	1.4	2.1	<0.01	7.9	117	15.7	7.8
	TF2	5.3	3.2	8.8	4.3	1.5	1.4	<0.01	7.8	123	15.8	7.6
Dry/ 2017	MA1	14.7	5.6	5.9	1.3	5.6	3.0	<0.01	7.5	128	15.9	7.1
	MA2	16.1	6.4	7.2	1.8	6.1	2.9	<0.01	8.9	145	15.2	7.0
	MA3	16.8	4.7	13.7	6.2	2.7	3.8	<0.01	7.9	220	17.1	6.3
	MA4	29.2	9.1	12.4	2.7	8.1	4.6	0.38	8.7	237	22.8	7.5
	MA5	18.9	9.4	20.5	3.2	5.9	2.0	0.49	8.0	240	22.6	6.9
	RI1	25.6	8.0	23.4	7.0	4.5	2.4	0.65	9.1	308	21.6	7.0
	RI2	23.8	7.6	22.6	6.3	4.4	2.3	0.64	9.0	283	25.4	7.0
	RI3	20.5	7.0	23.7	7.7	4.1	2.2	0.61	8.9	300	27.3	6.6
	RI4	26.9	11.3	25.8	7.3	5.8	2.6	0.69	9.0	333	27.7	6.9
	RI5	19.6	6.8	25.9	7.7	4.0	1.2	0.71	8.5	293	25.5	6.9
	RI6	20.2	6.8	25.6	7.1	3.9	2.0	0.61	8.5	286	25.7	6.5
	RI7	20.1	7.1	24.3	6.9	3.9	2.0	0.56	8.1	289	26.3	5.7
	S1	9.5	3.4	23.4	10.3	1.8	0.7	<0.01	8.7	247	23.5	6.9
	S2	14.0	4.1	26.3	6.9	2.9	0.8	<0.01	8.7	265	22.4	7.3
	S3	15.5	4.7	30.5	9.1	2.9	0.7	<0.01	8.9	293	22.5	7.4
	S4	15.9	4.9	25.2	9.6	3.0	1.2	<0.01	9.0	300	22.6	7.4
	S5	14.4	4.3	28.6	7.4	2.9	0.8	<0.01	8.8	293	21.0	7.4
	S6	15.1	4.5	24.0	7.8	3.4	1.4	<0.01	8.9	266	19.1	7.6
	S7	13.6	6.8	47.5	<0.04	2.2	1.9	0.75	8.7	381	24.0	6.9
	S8	15.1	4.0	24.0	0.0	3.0	0.8	0.65	8.6	268	22.5	7.3
	TF1	5.9	2.4	15.7	6.5	1.3	1.8	<0.01	8.2	163	15.7	7.7
	TF2	5.8	2.6	14.6	6.5	1.0	0.6	<0.01	8.6	155	16.6	7.6
	TF3	5.4	2.3	15.4	6.6	1.3	1.3	<0.01	8.3	158	18.0	7.4
Start wet/2018	MA1	16.2	7.9	6.0	1.5	9.8	5.0	<0.01	7.3	140	14.2	6.8
	MA2	19.5	9.6	8.7	0.0	13.3	6.2	0.09	7.0	188	16.5	6.1
	MA3	17.5	5.0	13.4	6.9	3.0	4.4	0.08	7.1	235	18.2	5.8
	MA4	25.8	10.2	12.7	2.5	10.5	6.3	0.14	8.3	242	21.0	6.8
	MA5	14.0	7.4	13.4	2.1	5.8	2.1	0.12	8.0	175	20.8	6.8
	RI1	25.7	7.9	15.8	4.8	5.8	3.9	0.17	8.4	270	21.4	7.2
	RI2	20.1	7.4	15.3	4.7	5.4	3.3	0.15	8.2	236	24.3	7.3
	RI3	17.5	7.9	15.2	4.6	4.4	3.3	0.16	8.0	233	23.5	7.3
	RI4	18.2	9.5	15.4	4.9	5.4	3.5	0.16	8.0	248	25.0	6.7
	RI5	15.8	7.3	15.8	4.5	4.3	3.3	0.13	8.2	213	25.0	7.5
	RI6	19.3	8.1	17.2	4.4	5.9	2.9	0.16	7.8	238	25.0	7.0
	RI7	19.6	8.6	19.8	4.7	5.1	2.9	0.19	7.5	246	25.4	5.8
	S1	7.7	3.7	15.5	7.1	2.1	1.5	0.11	8.1	182	21.6	7.5
	S2	14.2	4.9	19.3	6.4	3.9	1.1	0.13	8.1	247	21.0	7.5
	S3	15.9	5.8	18.4	7.9	4.8	1.8	0.13	8.3	276	21.1	7.6
	S4	16.3	5.9	16.9	8.0	4.8	2.7	0.14	8.3	280	21.3	7.5
	S5	13.9	4.9	19.6	6.6	4.0	1.1	0.14	8.2	288	20.0	7.2
	S6	17.3	5.7	17.5	7.8	5.1	1.6	0.14	8.7	278	20.0	7.6
	S7	8.3	4.7	21.9	8.2	2.7	2.0	0.16	8.3	253	23.4	7.2
	S8	13.6	5.0	16.4	5.7	3.4	2.7	0.16	8.1	212	23.6	7.4
	TF1	5.6	8.3	11.8	5.7	1.7	2.0	0.12	8.0	159	15.3	7.3
	TF2	6.7	3.6	13.1	7.5	1.0	0.5	0.38	8.2	177	16.8	7.3
	TF3	5.3	2.7	13.8	6.4	1.6	1.3	0.11	8.1	152	18.0	7.2
Peak wet/2018	MA1	8.1	4.0	2.6	0.8	7.7	3.8	0.05	7.2	136	16.0	7.6
	MA2	9.3	6.0	4.4	1.4	5.5	2.7	0.06	7.1	95	17.3	7.1
	MA3	10.1	5.2	3.7	1.5	5.9	3.1	0.24	7.6	140	21.1	7.0
	MA4	14.5	7.1	7.1	1.7	6.5	3.9	0.05	8.1	130	19.2	8.2
	MA5	14.9	5.4	9.3	3.0	4.7	1.6	0.05	7.9	121	19.5	8.0
	RI1	14.9	5.4	9.3	3.0	4.7	2.9	0.04	8.4	212	18.4	8.9
	RI2	6.5	3.4	5.0	1.2	4.1	1.9	0.04	8.1	168	21.5	8.9
	RI3	5.7	2.5	7.8	1.6	3.6	2.0	0.04	7.7	198	22.2	8.5
	RI4	9.7	5.0	7.4	1.7	4.7	2.5	0.04	8.0	174	21.5	8.7
	RI5	4.3	2.2	4.4	1.2	2.9	1.2	<0.01	7.8	178	21.6	9.1
	RI6	4.9	2.3	4.1	1.1	2.6	1.0	0.03	7.9	180	22.2	8.3
	RI7	12.5	5.2	11.6	2.6	3.4	2.0	0.04	7.0	182	22.8	4.4
	S1	5.7	2.2	12.1	3.8	4.3	3.8	0.03	7.8	148	22.2	8.5
	S2	5.7	2.0	6.8	2.0	3.3	1.2	0.04	7.9	217	22.5	8.7
	S3	7.3	3.0	9.6	2.8	3.2	2.4	0.03	8.3	207	20.8	9.0
	S4	7.4	3.6	7.1	2.5	4.0	2.1	<0.01	8.4	204	20.6	8.9
	S5	5.0	1.9	6.9	2.1	3.1	0.9	0.04	8.0	232	22.7	8.4
	S6	10.3	4.5	9.8	3.6	4.0	2.2	0.03	8.0	183	19.2	8.9
	S7	3.2	2.0	9.2	3.2	2.1	1.3	0.04	8.0	226	22.6	8.4
	S8	7.5	2.9	8.9	2.6	3.2	1.6	0.04	7.6	192	22.6	8.6
	TF1	3.4	2.1	8.9	4.0	1.3	1.5	0.03	7.5	96	16.4	8.5
	TF2	4.0	2.6	8.4	3.6	1.6	0.9	0.03	6.9	92	16.8	8.4
	TF3	3.5	1.9	9.1	4.4	1.4	1.3	0.03	7.3	103	18.3	8.5

## 2 Experimental Design, Materials and Methods

Field monitoring was undertaken in 23 spatially distributed sampling stations located in the main river channel and its two major tributaries (Fig. 1). The field monitoring strategy covered the key land use activities in the basin which includes: forests, commercial agriculture (tea, sugarcane, and horticulture), mixed agriculture (crops and livestock keeping), industrial areas, urban centres, and wetland. To capture seasonal trends in hydro-chemical and isotopic variables, field monitoring campaigns were conducted during four periods in a year, which are: the period between dry and wet season in March, referred here as ‘start wet’ (SW), the agriculturally productive wet period between May – July, referred as ‘peak wet’ (PW), during the ‘end of the wet season’ (EW) in September, and the dry season (D) in December. More details are described in [6]. A total of nine field monitoring campaigns were conducted between July 2016 and May 2018,which consisted of 2 SW, 3 PW, 2 EW, and 2 D seasons. During sampling, water was first pre-filtered onsite using 11 µm filters (Whatmann, GE Healthcare Life Sciences, Chicago, IL, USA), then transferred into 200 mL high density polyethylene (HDPE) bottles. As a necessary condition for samples destined for nitrate analysis, the samples were stored in an insulated cooler box containing ice cubes so as to keep a constant temperature of around 4 °C during transportation to the laboratory. However, samples for cation analysis were transferred into 100 mL HDPE bottles after pre-filtration, then acidified to pH 2 using diluted hydrochloric acid. The measurement of temperature (T), electrical conductivity (EC), pH, and dissolved oxygen (DO) was done *in situ* using a multi-parameter sensor (2FD47F-Multi3430, WTW, Germany). In addition, sampling was done for potential NO_3_^−^ end members (sewage, manure, inorganic fertilizers, precipitation, and soil nitrogen) for the determination of their δ^15^N‒, δ^18^O‒ NO_3_^−^ and δ^11^B‒B values. Sewage effluents were sampled from the inlet point of sewage treatment plants located in key towns like Kisumu, Kericho, Muhoroni, and Chemelil. Manure samples were taken from cow, goat and sheep droppings in the basin. Samples of the commonly used mineral fertilizers in the basin (CAN, DAP, NPK, urea) were purchased from farmers and suppliers. Rainfall samples were collected from stations located in Ahero, Kakamega and Kericho towns, while soil N samples were collected by filtering suspended soil sediments in river water using 11 µm filters (Whatmann, GE Healthcare Life Sciences, Chicago, IL, USA). Before laboratory analysis for δ^15^N‒ and δ^18^O‒NO_3_^−^,samples were filtered again through 0.45 µm membrane filters and stored frozen (-17°C). Laboratory analysis of the hydro-chemical parameters (Na^+^, K^+^, Ca^2+^, Mg^2+^, NO_3_^−^, NO_2_^−^, Cl^−^, and SO_4_^2−^) was carried out using an ion chromatograph (930 Compact IC Flex, Metrohm, Switzerland).

Analysis for δ^15^N‒ and δ^18^O‒NO_3_^−^ were carried out using the “Bacterial denitrification method” as adopted from [2], [3], [4]. The method allows for the simultaneous determination of δ^15^N and δ^18^O in N_2_O produced from the conversion of NO_3_^−^ by the denitrifying bacteria, *Pseudomonas aureofaciens. Pseudomonas aureofaciens* (recently reclassified as a strain of *Pseudomonas chlororaphis*) are ideal bacteria for simultaneous ^15^N and ^18^O analyses because they naturally lack N_2_O-reductase activity (the enzyme that reduces N_2_O to N_2_) and therefore provide information for both N and O isotopes. This method is applicable for seawater and freshwater samples at the natural-abundance level. Bacterial cultures were grown for 6–10 days in amended tryptic soy broth (TSB), divided into centrifuge tubes of 40 mL aliquots and centrifuged. After centrifugation, the supernatant was decanted, reserved and 4 mL of the TSB were pipetted back into the tubes to obtain a 10-fold concentration of bacteria. These tubes were then vortexed to ensure homogenized cultures and transferred as 2 × 2 mL aliquots into 20 mL headspace vials. The vials were crimp-sealed with Teflon-backed silicone septa. To ensure anaerobic conditions, a reduced blank effect and removal of N_2_O produced prior to sample injection, the headspace vials were purged with N_2_ gas for 3 hours. Samples of dissolved NO_3_^−^ (100 nmol) were then injected into the headspace vials and incubated overnight to allow for complete conversion of NO_3_^−^ to N_2_O. The next day, 0.1 mL of 10 N NaOH were injected into the headspace vials to stop bacterial activity and to scrub any CO_2_ gas in the vial which can interfere with the N_2_O measurement. The δ^15^N and δ^18^O analyses of the produced N_2_O were carried out using a trace gas preparation unit (ANCA TGII, SerCon, UK), coupled to an isotope ratio mass spectrometer (IRMS) (20-20, SerCon, UK). The N_2_O sample was flushed out of the sample vial using a double-hole needle on an auto-sampler. Water was removed using a combination of a nafion dryer and MgClO_4_ scrubber. The N_2_O was compressed onto a capillary column (CP-Poraplot Q 25 m, 0.32 mm id, 10 µm df, Varian, US) at 35°C by cryogenic trapping and focusing and subsequently analyzed by IRMS. Individual samples were ran in triplicate and the resultant isotope data is normally expressed as delta (δ) units in per mil (‰) notation, relative to international reference standards. This is expressed as:

(1) $$\delta_{sample}\left(\%\right)=\left[\frac{R_{sample}}{R_{standard}}-1\right]\times 1000$$

Where $R_{sample}\;$ and $R_{standard}$ are the ^15^N/^14^N or ^18^O/^16^O ratio of the sample and the standard for δ^15^N and δ^18^O, respectively. δ^15^N and δ^18^O values are reported relative to atmospheric N_2_ and Vienna Standard Mean Ocean Water (VSMOW) respectively. Three international reference standards, USGS32 (180.0 ± 1.0‰ for δ^15^N, 25.7 ± 0.4‰ for δ^18^O), USGS34 (-1.8 ± 0.2‰ for δ^15^N, -27.8 ± 0.4‰ for δ^18^O), and USGS35 (2.7 ± 0.2‰ for δ^15^N, 56.8 ± 0.3‰ for δ^18^O), were used to normalize the raw δ^15^N‒ and δ^18^O‒NO_3_^−^ values (based on a N_2_O reference gas tank) to the AIR and VSMOW scale. USGS32 and USGS34 were used for normalization of the δ^15^N value and USGS34 and USGS35 for the δ^18^O. NO_3_^−^ content in samples and references were harmonized (i.e. 20 nmol), this corrects for nonlinearity of the IRMS and blanks associated with the procedure. As a quality control measure, an in-house KNO_3_ laboratory standard (9.9‰ for δ^15^N, 24.3‰ for δ^18^O) was analyzed together with the samples. Measurement batches were only accepted if measured δ^15^N and δ^18^O values of the laboratory standard were within 0.4 and 0.5 ‰ of our accepted values, respectively. Incase standard deviation on replicate samples was higher than 0.3 and 0.4 for δ^15^N and δ^18^O, respectively, the sample was reanalyzed. More details about this technique are covered in [2,3].

The water analysis technique for B and δ^11^B was carried out as explained in [5]. Samples underwent a two-step chemical purification using Amberlite IRA-743-selective resin, a method adopted from [7]. First, the sample (pH∼7) was loaded on a Teflon PFA® column filled with 1 ml resin, previously cleaned with ultrapure water and 2N ultrapure NaOH. After cleaning the resin again with water and NaOH, the purified B was collected with 15 ml of sub-boiled HCl 2N. After neutralization of the HCl with Superpur NH_4_OH (20%), the purified B was loaded again on a small 100 ml resin Teflon PFA® column. B was collected with 2 ml of HCl 2N. An aliquot corresponding to 2 mg of B was then evaporated below 70°C with mannitol (C_6_H_8_(OH)_6_) in order to avoid B loss during evaporation [8]. The dry sample was loaded onto a tantalum (Ta) single filament with graphite (C), mannitol and cesium (Cs). δ^11^B values were then determined by measuring the Cs_2_BO_2_^+^ ion [9,10] by a thermal ionization mass spectrometer. The analysis was ran in dynamic mode by switching between masses 308 and 309. Each analysis corresponded to 10 blocks of 10 ratios and every sample was ran twice. Total B blank was less than 10 ng, corresponding to a maximum contribution of 0.2%, which is negligible. Purification of seawater (IAEA-B1) was regularly conducted in the same way. Its purpose is to check for possible chemical fractionation which might be occasioned by an uncompleted recovery of B, and to evaluate the accuracy and reproducibility of the overall procedure [11]. Reproducibility was obtained by repeated measurements of the NBS951, and the accuracy was controlled with the analysis of the IAEA-B1 seawater standard (δ^11^B=38.6±1.7‰). Similar to N and O, B isotope ratios were expressed in delta (δ) units and a per mil (‰) notation relative to an international standard, NBS951.

## Ethics Statement

The authors agree upon the standards of the expected ethical behaviour

## Declaration of Competing Interest

The authors declare that they have no known competing financial interests or personal relationships which have or could be perceived to have influenced the work reported in this article.
